# Soil Nitrogen Application Regulates Photosynthetic Limitations in Plants: Insights from a Controlled Experiment

**DOI:** 10.3390/plants15213309

**Published:** 2026-10-26

**Authors:** Lina Gao, Qihui Zuo, Huiying Xiang, Siyu Tan, Kai Zhu

**Affiliations:** 1 College of Veterinary Medicine, Shanxi Agricultural University, Jinzhong 030801, China; 2 College of Resources and Environment, Shanxi Agricultural University, Jinzhong 030801, China; 3 State Key Laboratory of Sustainable Dryland Agriculture, Shanxi Agricultural University, Taiyuan 030031, China; 4 Soil Health Laboratory in Shanxi Province, Taiyuan 030031, China

**Keywords:** *Glycine max*, urea fertilization, mesophyll conductance, leaf nitrogen, Rubisco activity

## Abstract

Soil nitrogen (N) availability modulates the relative magnitudes and dominant roles of stomatal, mesophyll, and biochemical limitations to photosynthesis (denoted as *l*_sc_, *l*_m_, and *l*_b_, respectively); however, the dynamic changes in these factors and their relative contributions under varying N application levels remain controversial. In this study, we quantified the responses of photosynthetic limitations in soybeans subjected to four N application levels (0, 7.5, 15.0, and 22.5 g urea m^−2^). Moderate N addition (≤15.0 g urea m^−2^) increased *l*_sc_ and *l*_b_ but decreased *l*_m_, whereas excessive N addition (22.5 g urea m^−2^) caused a rapid increase in *l*_m_, exceeding both *l*_sc_ and *l*_b_. *l*_m_ was negatively correlated with mesophyll conductance (*g*_m_) and net photosynthetic rate (*A*_n_), whereas *l*_b_ was positively correlated with *A*_n_. Changes in photosynthetic limitation were accompanied by increased chlorophyll and leaf N contents and increased activities of aquaporins (AQPs), carbonic anhydrase (CA), and Rubisco under moderate N addition. These results indicated that the relative contribution of *l*_m_ to the total limitation (denoted as *MC*_L_) decreased under moderate N addition but increased sharply under high N addition, exceeding the contributions of *l*_sc_ and *l*_b_ (denoted as *SC*_L_ and *B*_L_, respectively). Under an excessive N supply of 22.5 g urea m^−2^, *MC*_L_ was the highest among the three components, although this ranking should be interpreted considering the reference-state dependence of the limitation framework. This study provides a basis for rational N fertilization in soybean production.

## 1. Introduction

Photosynthesis is the core physiological process of plant carbon assimilation and directly affects the growth, development, and yield of plants [1,2]. The efficiency of photosynthesis is regulated by multiple internal and external factors, among which the availability of nutrients, especially nitrogen (N), is among the most critical [3]. N is a key nutrient element for soybean growth, development, and photosynthetic processes; it directly affects Rubisco activity, stomatal development, mesophyll cell structure, and other traits, thereby regulating the partitioning of photosynthetic limitation components [4,5,6]. As a typical legume crop, soybean can establish symbioses with rhizobia to acquire atmospheric N via biological N fixation, whereas high mineral-N input typically represses nodule initiation and reduces symbiotic N-fixation capacity [7,8]. Nevertheless, the present study focuses mainly on the physiological responses of leaf photosynthetic limitations to externally supplied mineral N.

The limitations to photosynthesis in plants can be divided into three main categories: stomatal limitation (*l*_sc_), mesophyll limitation (*l*_m_), and biochemical limitation (*l*_b_) [9]. *l*_sc_ results from reduced stomatal conductance (*g*_sc_), which restricts CO_2_ entry into leaves. Subsequently, *l*_m_ is governed by mesophyll conductance (*g*_m_), which controls CO_2_ diffusion from intercellular airspaces to chloroplast carboxylation sites. Ultimately, *l*_b_ results from decreased activity of photosynthesis-related enzymes (e.g., Rubisco) and impaired product synthesis, thereby constraining CO_2_ fixation efficiency [10,11]. Photosynthetic limitation analysis serves as a central tool for determining the mechanism by which N regulates photosynthesis. Identifying the dominant limiting factor in the presence of different amounts of N is highly important for precise N management in soybean production. Previous studies have shown that N application can increase plant photosynthetic capacity by increasing chlorophyll content and photosynthesis-related enzyme activity [12,13,14], but the dynamic changes in the three types of photosynthetic limitations upon the application of different amounts of N and their relative contributions to photosynthesis are still controversial.

Both N deficiency and abundance can reduce the photosynthetic rate [12]. Most previous studies on N application and photosynthesis have focused on changes in the photosynthetic rate and related parameters [6,13,15], whereas quantitative analyses of photosynthetic limitations and of the mechanisms regulating these limitations remain scarce. Consequently, the reported dominant limitation under N deficiency is still debated: some studies identified *l*_sc_ as the main constraint [15], whereas others emphasized *l*_m_ or *l*_b_ [16,17]. In our latest study, Zuo et al. (2026) showed that enhanced biochemical metabolism under N addition was the dominant factor improving intraleaf CO_2_ diffusion in soybean leaves [18]; however, it remains unclear whether this enhancement mainly reflects increased photosynthetic carbon assimilation after soil N addition. In addition, excessive N application can lead to nutrient imbalance [19,20], which may cause a decrease in photosynthetic efficiency, but the specific type and degree of photosynthetic limitation under excessive N still need clarification.

Accordingly, the aim of this study was to quantify the dynamic changes in the photosynthetic limitation components (*l*_sc_, *l*_m_, and *l*_b_) of soybean when different amounts of N are added and identify the dominant limiting factor responsible for reduced photosynthesis under varied N conditions. We hypothesized that *l*_sc_ and *l*_b_ are the dominant constraints that lead to the increase in photosynthesis under low N conditions, that no obvious dominant limitation occurs under only moderate N conditions, and that *l*_m_ prevails only under high N conditions. The findings of this study will fill the research gap concerning the relationship between N application and photosynthetic limitations in soybean, elucidate the intrinsic mechanism through which N regulates soybean photosynthesis, and provide a theoretical basis and technical reference for the precise application of N fertilizer and for improving photosynthetic efficiency and grain yield in soybean.

## 2. Materials and Methods

### 2.1. Plant Material

Seedlings of the disease- and drought-resistant soybean (*Glycine max* L.) cultivar ‘Jindou 23’ (state-certified No. 2001011) were planted in individual pots at the Experimental Station of the College of Resources and Environment, Shanxi Agricultural University, Taigu, Shanxi, China (37°43′ N, 112°56′ E; 791 m a.s.l.) in late March 2021, with three replicate pots per treatment and two seedlings per pot. The pots were 12.2 L in volume, with a height of 24.0 cm and a diameter of 31.0 cm, and were filled with 7.4 L of soil. The initial soil total N content was 0.58 g kg^−1^, and the phosphorus content was 0.88 g kg^−1^. The baseline soil N content was determined using the Kjeldahl digestion method on air-dried soil samples passed through a 2 mm sieve prior to the addition of N and is expressed as total soil N content rather than being estimated from soil organic matter. The plant roots were blocked from the outside soil by pedestals placed under the pots. The relevant morphological parameters of the soybean seedlings when gas exchange was measured are shown in Table 1.

### 2.2. Experimental Design

Four N application levels, 0 (no N addition, CK), 7.5 (low N addition, LN), 15.0 (medium N addition, MN) and 22.5 g urea m^−2^ (high N addition, HN), were used to simulate the ecological effects under varying intensities of atmospheric N deposition. Urea solutions with different concentrations of N were sprayed evenly onto the surface of the rhizosphere soil in the different treatment groups. The plants in the CK group were sprayed with the same volume of water. Three replicates (i.e., three potted plants) were included in each treatment group, with two seedlings retained per pot. Six fully expanded mature leaves (three leaves from each individual plant) were selected per pot for measurements of photosynthetic and physiological parameters, and the mean values were taken as the replicate values for that potted plant. All the potted slings were thoroughly watered daily to avoid water deficit.

### 2.3. Analysis of Photosynthesis Limitations

Gas exchange and chlorophyll fluorescence on newly and fully expanded, sun-exposed leaves were simultaneously measured using an open-flow gas exchange system (Li-6400XT; LI-COR Inc., Lincoln, NE, USA) equipped with an integrated fluorescence leaf chamber (Li-6400-40; LI-COR) from 12 to 20 July 2022. During this period, the leaves were fully light-adapted under a saturated photosynthetically active photon flux density (PPFD) of 1200 µmol m^−2^ s^−1^ provided by Li-6400 with 10:90 blue:red light for 30 min; this saturating PPFD was selected on the basis of light response curves for soybean and is consistent with that used in previous studies [5,21]. The leaf temperature, relative humidity and CO_2_ concentration in the leaf chamber were maintained at 25 °C, 60 ± 5% and 400 μmol CO_2_ mol^−1^, respectively, with a CO_2_ mixture. These environmental settings follow the standard configuration for photosynthetic limitation analysis [22,23]. In addition, the gas flow rate was controlled at 300 μmol s^−1^ to ensure adequate gas exchange. After stabilization to a steady state, gas exchange parameters (i.e., net photosynthetic rate (*A*_n_, µmol CO_2_ m^−2^ s^−1^), intercellular CO_2_ concentration (*C*_i_, µmol mol^−1^)), steady-state fluorescence (*F*_s_) and maximum fluorescence (*F*_m_′) were recorded.

In accordance with Grassi and Magnani (2005) and Wang et al. (2018) [22,23], the relative changes in light-saturated assimilation are sequentially expressed in terms of parallel relative changes in *g*_sc_ and *g*_m_ and in biochemical capacity (e.g., in maximum carboxylation rate, *V*_cmax_) as follows:

(1) $$\frac{dA_{n}}{A_{n}}={SC}_{L}+{MC}_{L}+B_{L}=l_{sc}\cdot \frac{dg_{sc}}{g_{sc}}+l_{m}\cdot \frac{dg_{m}}{g_{m}}+l_{b}\cdot \frac{dV_{cmax}}{V_{cmax}}$$

$$l_{sc}=\frac{g_{tot}/g_{sc}\cdot \partial A_{n}/\partial C_{c}}{g_{t}+\partial A_{n}/\partial C_{c}}$$

(2) $$l_{m}=\frac{g_{tot}/g_{m}\cdot \partial A_{n}/\partial C_{c}}{g_{t}+\partial A_{n}/\partial C_{c}}$$

$$l_{b}=\frac{g_{tot}}{g_{tot}+\partial A_{n}/\partial C_{c}}$$

where *l*_sc_, *l*_m_ and *l*_b_ are the limitations imposed by *g*_sc_, *g*_m_ and biochemical capacity, respectively (0 < *l*_i_ < 1, i = sc, m, b); *SC*_L_, *MC*_L_ and *B*_L_ are the relative contributions of *g*_sc_ and *g*_m_ and biochemical capacity to d*A*_n_/*A*_n_, respectively; and the sum of contributions due to *g*_sc_ and *g*_m_ is defined as the diffusional limitation (*D*_L_ = *SC*_L_ + *MC*_L_), whereas the sum of *g*_m_ and biochemical capacity is defined as the nonstomatal limitation (*NS*_L_ = *MC*_L_ + *B*_L_) [22]. Within the Grassi–Magnani framework, limitation coefficients (*SC*_L_, *MC*_L_ and *B*_L_) represent the relative contribution of each component (*g*_sc_ and *g*_m_ and *V*_cmax_) to the relative change in *A*_n_ with respect to a defined reference state and are not absolute measures of constraint intensity. In the current study, *V*_cmax_ was replaced with *J*_f_, which is coupled with *V*_cmax_ [23,24,25,26], and the fitted photosynthesis parameters at *Ψ*_pd_ = −0.25 MPa (CK) were used as reference values [23]. Thus,

(3) $$\frac{dx}{x}=\frac{x_{ref}-x}{x_{ref}}=\frac{x_{0.25}-x}{x_{0.25}}$$

where *x* represents the fitted *g*_sc_, *g*_m_ or *J*_f_ and *x*_0.25_ (e.g., *x*_ref_) represents the *x* value at *Ψ*_pd_ = −0.25 MPa (CK).

Because *A*_n_-*C*_i_ curves were not measured, *V*_cmax_ and *J*_max_ were derived from light-response gas-exchange-fluorescence data, and triose phosphate utilization (TPU) could not be estimated. Importantly, biochemical limitation (*l*_b_) is also modulated by TPU in addition to *V*_cmax_ and *J*_max_, which represents a methodological limitation of the current analysis.

*g*_sc_ was determined by the following Formula (4):

(4) $$g_{sc}=g_{sw}/1.6$$

where *g*_sw_ is the stomatal conductance to water vapour (mol (H_2_O) m^−2^ s^−1^), which was directly obtained from gas exchange measurements, and 1.6 is the ratio of the diffusivities of CO_2_ and water in air.

*g*_m_ was calculated using the ‘variable *J* method’ described by Harley et al. (1992) [27]:

(5) $$g_{m}=\frac{A_{n}}{C_{i}-\frac{\Gamma^{*}\left(J_{f}+8\left(A_{n}+R_{d}\right)\right)}{J_{f}-4(A_{n}+R_{d})}}$$

where *J*_f_ is the photosynthetic electron transport rate, Γ* represents the CO_2_ compensation point in the absence of respiration (µmol mol^−1^), and *R*_d_ represents the mitochondrial respiration in the light (µmol m^−2^ s^−1^).

In this study, *J*_f_ was calculated as follows:

(6) $$J_{f}=\Phi_{PSII}\cdot PPFD\cdot \alpha \beta$$

(7) $$\Phi_{PSII}=\frac{\left(F_{m}^{\prime }-F_{s}\right)}{F_{m}^{\prime }}$$

where *Φ*_PSII_ is the actual photochemical efficiency of photosystem II, which is calculated according to Genty et al. (1989) [28] using Formula (7). *α* is the total leaf absorptance, and *β* is the partitioning of absorbed quantum between PS II and PS I. In this study, *αβ* was corrected as described by Valentini et al. (1995) [29], and its values for different N treatments are shown in Table 2.

Γ* and *R*_d_ were measured using the Laisk method [30]. In theory, they intersect with each other at a point, and the intersection points at the *x*- and *y*-axes are considered to indicate the intercellular CO_2_ partial pressure (*C*_i_*) and *R*_d_, respectively. In practice, three CO_2_ response curves under low light and low CO_2_ concentrations obtained by varying CO_2_ concentrations from 150 to 40 µmol CO_2_ mol^−1^ under three PPFDs (150, 100 and 50 µmol m^−2^ s^−1^) formed a triangular range rather than a single point [31], and *C*_i_^*^ and *R*_d_ were calculated as the barycentre of the triangle formed by the intersection of the three lines on the *x*- and *y*-axes, respectively, in this study. The value of Γ* was calculated using the following Formula (8) [32]:

(8) $$\Gamma^{*}=C_{i}^{*}+\frac{R_{d}}{g_{m}}$$

*g*_tot_ was the total conductance to CO_2_ and was calculated using Formula (9):

(9) $$g_{tot}=\frac{1}{\frac{1}{g_{sc}}+\frac{1}{g_{m}}}$$

Notably, the values for biochemical and anatomical traits, including aquaporin (AQP) activity, carbonic anhydrase (CA) activity, Rubisco activity, cell wall thickness (*T*_cw_), surface area of mesophyll cells exposed to intercellular space per unit of leaf area (*S*_m_), chloroplast surface area facing the intercellular space per unit of leaf area (*S*_c_), liquid-phase conductance to CO_2_ (*g*_liq_), and *J*_f_, used in this study were obtained from our companion study in Zuo et al. (2026) [18], which was conducted using the same experimental system and plant material. The present study focused on the quantitative analysis of photosynthetic limitations, whereas the study by Zuo et al. (2026) [18] focused on internal leaf CO_2_ diffusion and biochemical metabolism. Together, these two studies provide a complementary understanding of how soil N supply regulates soybean photosynthesis.

Notably, the limitation coefficients *SC*_L_, *MC*_L_ and *B*_L_ in the Grassi–Magnani framework are defined relative to a chosen reference state. In this study, the CK treatment (0 g urea m^−2^, *Ψ*_pd_ = −0.25 MPa) was used as the reference. Consequently, all the limitation values are relative to this reference and should not be interpreted as absolute constraint intensities. A reference-dependence sensitivity analysis was performed to assess the robustness of the inferred limitation rankings (see Section 3.4).

### 2.4. Determination of Chlorophyll Content

The chlorophyll content was determined using the Arnon method [33]. Fresh leaf fragments (0.1 g) with the veins removed were weighed and placed in a mortar. A small amount of quartz sand and calcium carbonate, along with 2 mL of 80% acetone, was added, and the sample was ground thoroughly into a homogenate, which was then transferred to a 25 mL brown volumetric flask. The mortar, pestle, and residue were rinsed 2–3 times with a small volume of 80% acetone, the rinses were combined, and the volume was made up to 25 mL with 80% acetone and mixed well. The samples were extracted in the dark for 12 h, followed by centrifugation at 4 °C and 8000 r min^−1^ for 10 min, after which the supernatant was collected. Using 80% acetone as the blank, the absorbance was measured at 663 nm and 645 nm using UV-Vis/Vis Spectrophotometers (Ther-moFisher Scientific, Waltham, MA, USA). The chlorophyll content was calculated using the following formulas and is expressed as mg g^−1^ FW: Chl a = 12.7A_663_ − 2.69A_645_; Chl b = 22.9A_645_ − 4.68A_663_; and Chl total = 8.02A_663_ + 20.21A_645_.

### 2.5. Measurement of Leaf N Content

Fully expanded and healthy functional leaves were randomly selected during the gas exchange measurement period and immediately cleaned with deionized water to remove surface dust and soil residues. The samples were subsequently oven-dried at 75 °C for 24 h until a constant weight was achieved to eliminate residual moisture and then thoroughly ground into a fine and homogeneous powder using an oscillating mixer mill (MM400; Retsch, Düsseldorf, Germany). Approximately 5.0 mg of each leaf sample was accurately weighed and loaded into a C–N elemental analyser (Elementar Vario MACRO, Element, Langenselbold, Germany) to determine the leaf N content (LNC). The obtained N content was calculated and is expressed on a dry weight basis, and replicate measurements (*n* = 3) were performed to guarantee the accuracy and reproducibility of the experimental data.

### 2.6. Statistical Analysis

Normality and homogeneity of variance were confirmed prior to parametric analysis. One-way ANOVA was performed using IBM SPSS Statistics 29.0 (SPSS, Inc., Chicago, IL, USA) to determine differences in *l*_sc_, *l*_m_ and *l*_b_ among the different N treatment groups, and means were compared using the least significant difference (LSD) multiple comparison test. In addition, regression analysis was used to describe the response of each limitation component to the gradient of added N, with the N dose treated as a continuous variable; both linear and quadratic models were fitted, and the best-fitting model was selected on the basis of R^2^ and the significance level. Pearson correlation analysis and linear regression analysis were subsequently performed to examine the relationships between photosynthetic limitation components (*l*_sc_, *l*_m_ and *l*_b_) and physiological traits (*g*_m_ and *A*_n_), as well as between *A*_n_ and the limitation components. Correlation coefficients (r), regression parameters and their significance levels were calculated. A sensitivity analysis was subsequently conducted to evaluate the effects of the limitation components on the photosynthetic carbon assimilation capacity, which was assessed as *A*_n_ and *V*_cmax_. Differences were considered statistically significant at *p* < 0.05. All figures were generated using OriginPro 2021 (OriginLab Corporation, Northampton, MA, USA).

## 3. Results

### 3.1. Quantitative Changes in Photosynthetic Limitation Components

**Figure 1:**
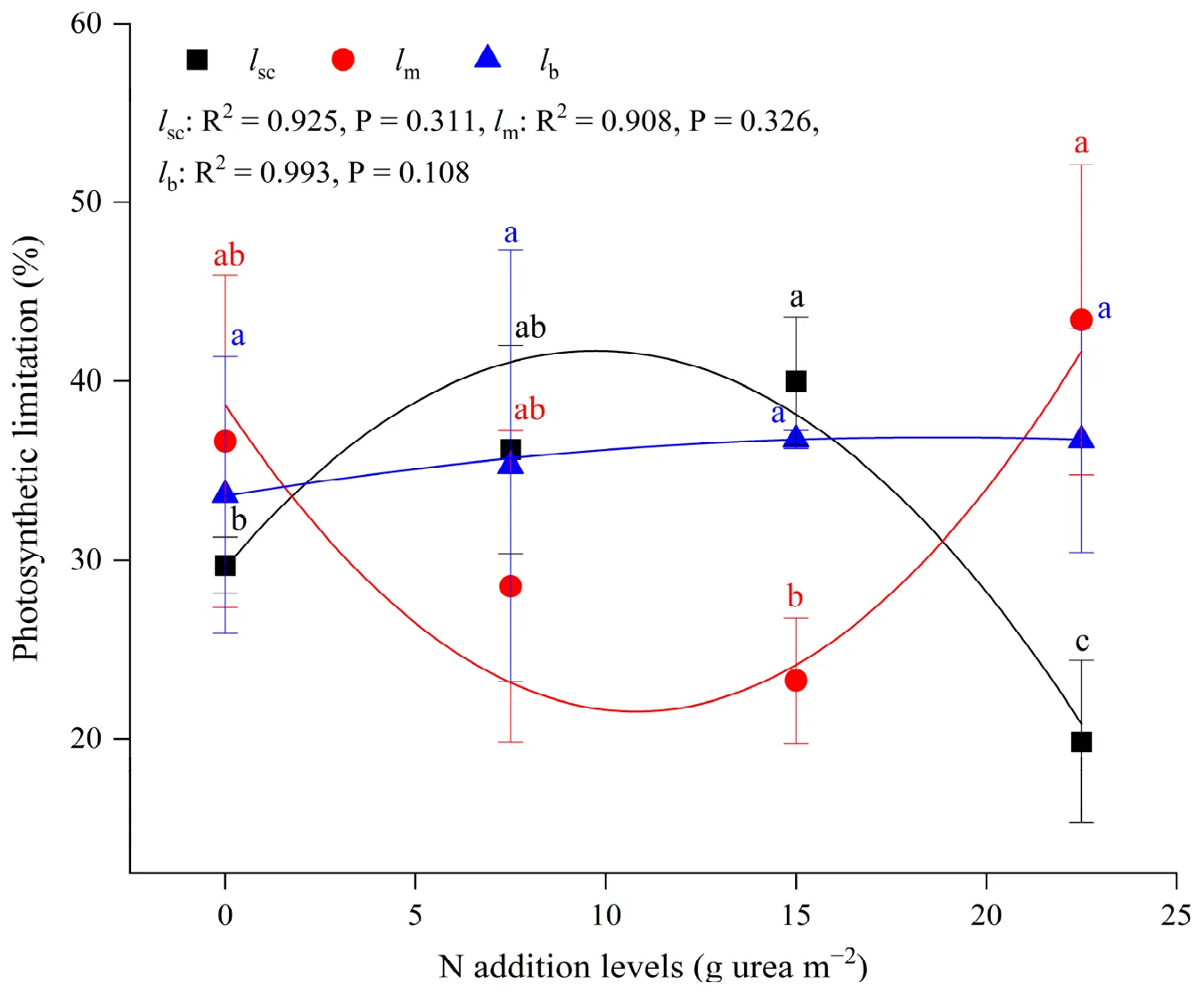
Response of photosynthetic limitation components to soil N application levels. *l*_sc_, stomatal limitation; *l*_m_, mesophyll limitation; *l*_b_, biochemical limitation. The values are the means ± SDs (*n* = 3). Different lowercase letters (a, b, and c) denote significant differences among the N addition treatment groups at *p* < 0.05. Regression curves were fitted against the N application level as a continuous variable: *l*_sc_, quadratic model, *R*^2^ = 0.925, *p* = 0.311; *l*_m_, quadratic model, *R*^2^ = 0.908, *p* = 0.326; *l*_b_, quadratic model, *R*^2^ = 0.993, *p* = 0.108. CK, control; LN, low N addition; MN, medium N addition; HN, high N addition.

The responses of the three photosynthetic limitation indices (*l*_sc_, *l*_m_, and *l*_b_) to the gradient of added N are shown in Figure 1. One-way ANOVA revealed significant differences among the N treatment groups in terms of *l*_sc_ (*F* = 11.261; *p* = 0.003), *l*_m_ (*F* = 3.652; *p* = 0.063), and *l*_b_ (*F* = 0.104; *p* = 0.955). When the N dose was treated as a continuous variable, quadratic models provided the best fit for all three components, although none reached statistical significance (*l*_sc_: *R*^2^ = 0.925, *p* = 0.311; *l*_m_: *R*^2^ = 0.908, *p* = 0.326; *l*_b_: *R*^2^ = 0.993, *p* = 0.108; Figure 1), likely owing to the limited number of N levels (*n* = 4) and the resulting low statistical power. Despite the nonsignificant regressions, *l*_sc_ increased from the CK group to the MN group and then decreased in the HN group; *l*_m_ exhibited a U-shaped trend, reaching a minimum at 10.9 g urea m^−2^ and showing a sharp increase at 22.5 g urea m^−2^; and *l*_b_ gradually increased. At the highest N dose, *l*_m_ exceeded both *l*_sc_ and *l*_b_, although this difference was not statistically significant for all pairwise comparisons, indicating a shift in the dominant limitation from mesophyll to stomatal and biochemical constraints as the N supply increased.

### 3.2. Quantitative Changes in the Relative Contribution of Photosynthetic Limitation Components to Photosynthetic Carbon Assimilation

**Figure 2:**
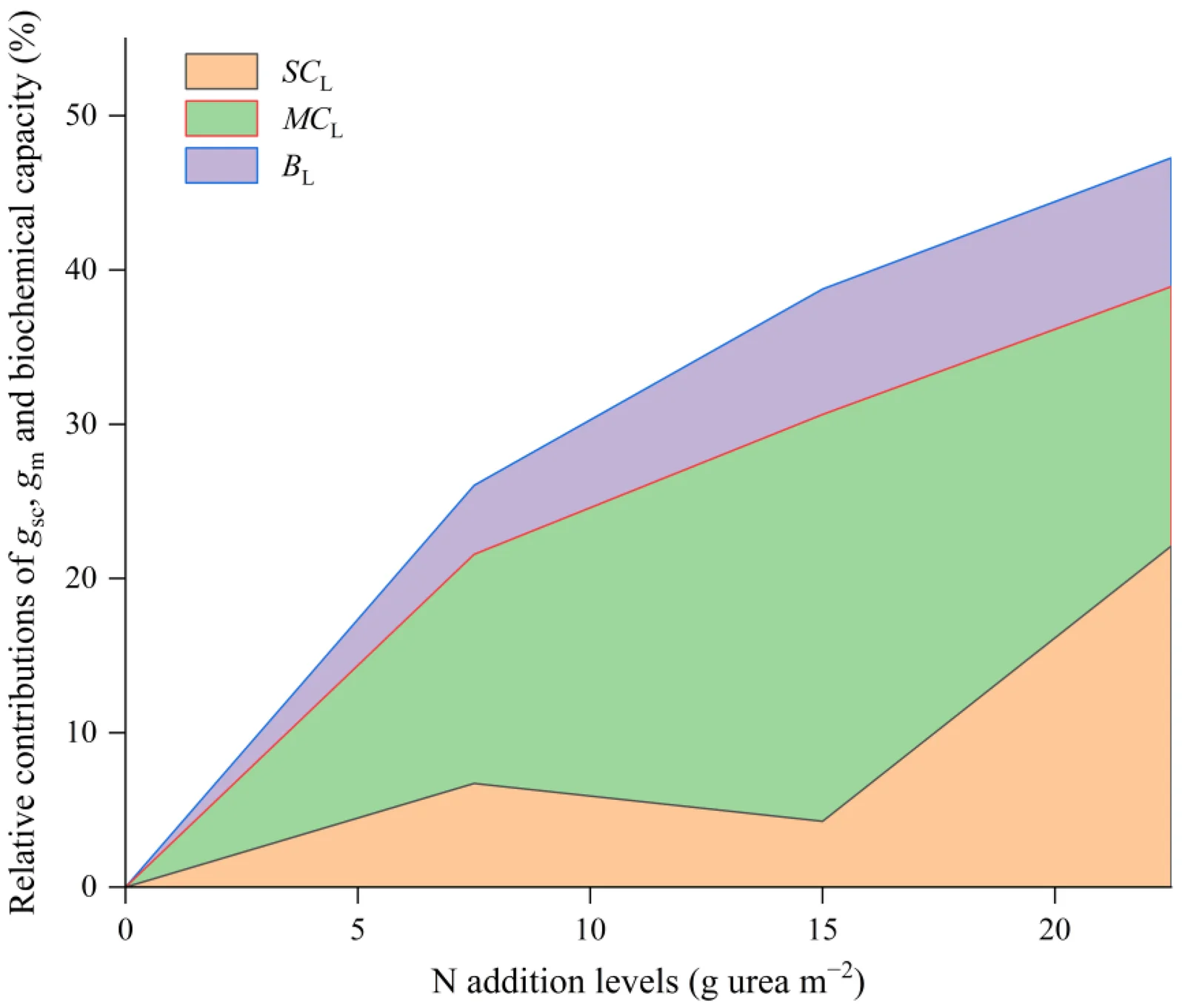
Relative contribution of photosynthetic limitation components to photosynthetic carbon assimilation.

We quantified the relative contributions of photosynthetic limitation components (*SC*_L_, *MC*_L_ and *B*_L_) to photosynthetic carbon assimilation with increasing N application levels (Figure 2). Overall, the three indices responded differently to increasing N supply: *SC*_L_ exhibited a biphasic response, *MC*_L_ remained the largest single component across most of the gradient, and *B*_L_ increased gradually but stayed comparatively small. Specifically, *SC*_L_ was negligible at low N levels (0%) and peaked at approximately 22% under excess N. *MC*_L_ ranged from 34% to 39% relative to the CK treatment across the N gradient, with *g*_m_ accounting for 60–70% of the total limitation. *B*_L_ increased gradually from 1% to 9% with increasing N availability. Consequently, the total photosynthetic limitation nearly doubled from 35% (control) to 70% at the highest N level. Our findings indicate that *g*_m_ was the core bottleneck of photosynthesis under N addition, whereas excess N simultaneously strengthened stomatal and biochemical constraints, providing a mechanistic basis for optimizing N management to improve plant photosynthetic efficiency.

### 3.3. Correlations Between Limitation Components and Physiological Traits

**Figure 3:**
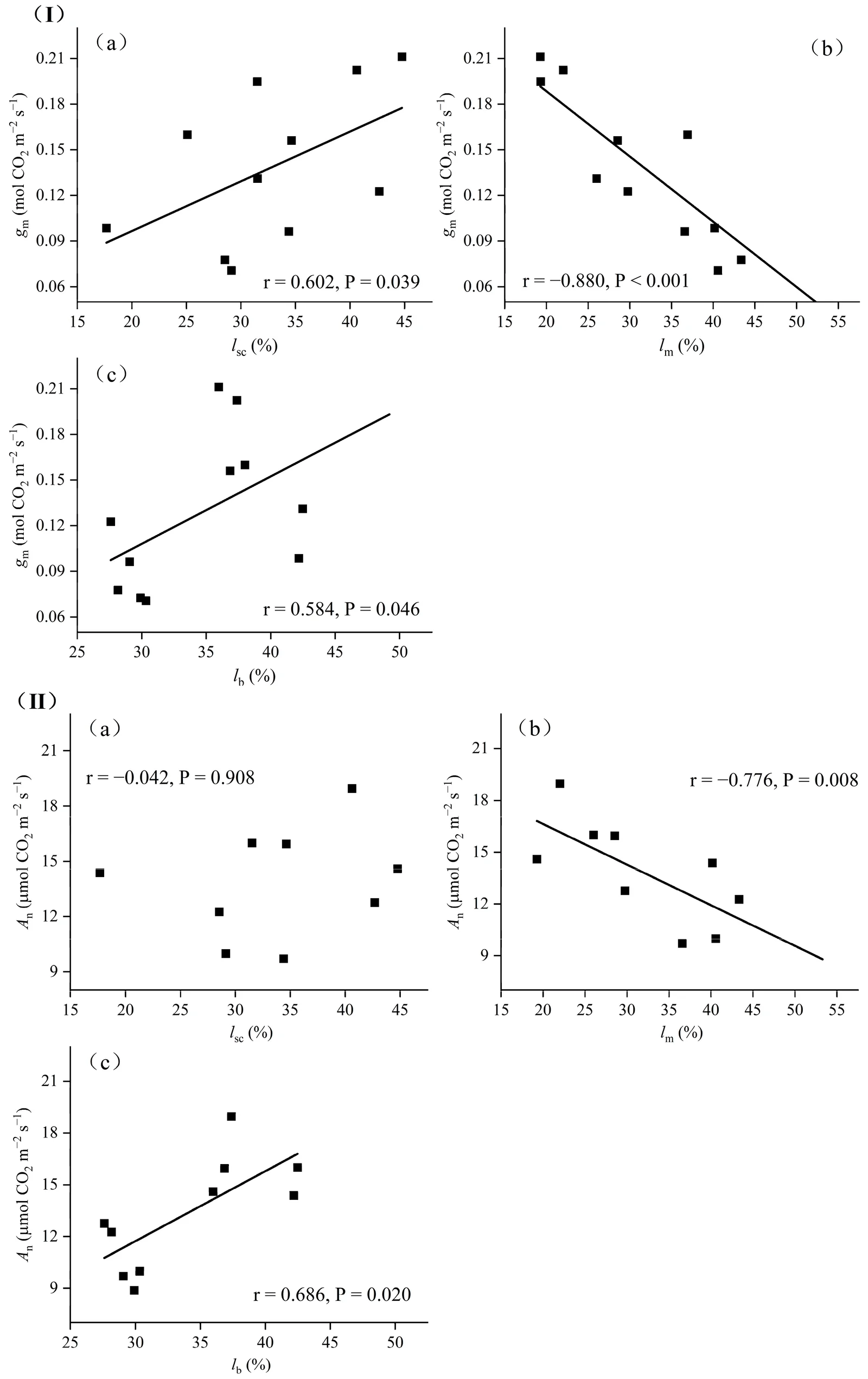
Correlations between *g*_m_ (**I**) and *A*_n_ (**II**) and limitation components (*l*_sc_, *l*_m_ and *l*_b_). *l*_sc_, stomatal limitation, *l*_m_, mesophyll limitation, *l*_b_, biochemical limitation. The fitting lines are shown at the level of *p* < 0.05. I-a, b, c are correlations between *g*_m_ and *l*_sc_, *l*_m_ and *l*_b_, respectively; II-a, b, c are correlations between *A*_n_ and *l*_sc_, *l*_m_ and *l*_b_, respectively.

We first examined the relationships between *g*_m_ and the three limitation components, and then assessed how each component was related to *A*_n_ (Figure 3). As shown in Figure 3I, *g*_m_ was significantly positively correlated with *l*_sc_ (r = 0.602, *p* = 0.039) and *l*_b_ (r = 0.584, *p* = 0.046) but extremely significantly negatively correlated with *l*_m_ (r = −0.880, *p* < 0.001). *l*_m_ acted as the dominant negative regulator that directly constrained *g*_m_, whereas *l*_sc_ and *l*_b_ exerted positive effects on *g*_m_, probably via a compensatory mechanism in which *g*_m_ was increased to alleviate stomatal/cuticular and biochemical constraints on photosynthesis. Thus, *g*_m_ appeared to be co-regulated by these three limitation components, with *l*_m_ as the decisive factor and *l*_sc_ and *l*_b_ as compensatory positive drivers.

As illustrated in Figure 3II, linear regression analyses revealed divergent associations between *A*_n_ and the three photosynthetic limitation components. No statistically significant linear correlation was observed between *A*_n_ and *l*_sc_ (*p* > 0.05), indicating that *g*_sc_ did not have a meaningful linear effect on *A*_n_ across the soil N supply range of 0–22.5 g urea m^−2^. Conversely, a strong and statistically significant negative linear correlation was observed between *A*_n_ and *l*_m_ (r = −0.776, *p* = 0.008), demonstrating that elevated mesophyll limitation was associated with a pronounced reduction in photosynthetic CO_2_ assimilation, with *l*_m_ explaining a large proportion of the variance in *A*_n_. Furthermore, a moderate and statistically significant positive linear correlation was identified between *A*_n_ and *l*_b_ (r = 0.686, *p* = 0.020), indicating that a higher biochemical limitation was linked to increased *A*_n_, likely reflecting compensatory physiological adjustment. Collectively, these findings indicated that *l*_m_ acted as the primary negative linear predictor of *A*_n_, *l*_b_ functioned as a significant positive linear predictor, and *l*_sc_ had no significant linear predictive power for the net photosynthetic rate, a pattern consistent with the regulatory dynamics of *g*_m_ described above.

### 3.4. Reference-Dependent Sensitivity Analysis of the Relative Contribution of Photosynthetic Limitation Components to Photosynthetic Carbon Assimilation

The Grassi–Magnani limitation framework is reference-state dependent. To test the robustness of the limitation-component rankings, the LN and MN treatments were separately adopted as alternative reference states to recalculate *SC*_L_, *MC*_L_ and *B*_L_ (Table 3).

When the LN treatment was used as the reference, *MC*_L_ (12.01 ± 2.43%) > *SC*_L_ (5.92 ± 1.15%) > *B*_L_ (4.61 ± 1.30%) for the CK treatment, whereas *SC*_L_ (25.58 ± 2.90%) > *MC*_L_ (21.90 ± 2.95%) > *B*_L_ (11.55 ± 1.47%) for the HN treatment. When the MN treatment was used as the reference, *MC*_L_ was still the largest component in the CK group (19.29 ± 2.09%), and *SC*_L_ (22.52 ± 2.27%) and *MC*_L_ (19.18 ± 2.13%) were comparable in the HN group, with BL remaining the minor contributor (5.73 ± 0.87%). The HN treatment consistently produced the highest values for all three contribution indices across reference states.

Comparison with the original results in which the CK treatment was used as the reference (Section 3.2, where *MC*_L_ dominated the total limitation) showed that switching reference states altered the numerical order of *SC*_L_ and *MC*_L_, especially for the HN treatment. Nevertheless, the sum of the diffusional limitations (*SC*_L_ + *MC*_L_) always constituted the major fraction of the total photosynthetic limitation, whereas *B*_L_ remained the smallest component regardless of reference selection. These results confirm that individual limitation rankings are reference-dependent rather than absolute; however, the qualitative conclusion that diffusional limitations dominate photosynthetic constraints under varying soil N levels remains robust.

### 3.5. Sensitivity Analysis of the Limiting Components of Photosynthetic Carbon Assimilation

On the basis of the linear regression analysis between the photosynthetic limitation components (*l*_sc_, *l*_m_ and *l*_b_) and photosynthetic carbon assimilation (*A*_n_ and *V*_cmax_) presented in Table 4, the VIF for all the photosynthetic limitation factors was greater than 1.000, indicating the absence of multicollinearity among the independent variables. For *A*_n_ (left panel), *l*_b_ had the largest standardized coefficient (beta = 0.568), followed by *l*_sc_, with a moderate positive effect (beta = 0.261, *p* = 0.357), whereas *l*_m_ exhibited a negative standardized coefficient (beta = −0.515) that was not statistically significant (*p* = 0.087). The overall model *F*-statistics for *l*_sc_ and *l*_b_ (*F* = 2.519, df = 11) and for *l*_m_ (*F* = 3.603, df = 11) further suggested limited explanatory power. For *V*_cmax_ (right panel), all three factors showed weak and nonsignificant effects, with *l*_b_ having a weak negative influence (beta = −0.279, *p* = 0.406), whereas *l*_sc_ (beta = 0.075, *p* = 0.820) and *l*_m_ (beta = 0.097, *p* = 0.765) had negligible positive effects. Similarly, the model F-values were also low (*F* = 0.435 for *l*_sc_ and *l*_b_; *F* = 0.095 for *l*_m_), confirming that the relationships were not significant. These results suggested that all three limitation components might contribute to variation in *A*_n_, with *l*_b_ and *l*_m_ showing marginal trends (*p* ≈ 0.06 and 0.09, respectively) and *l*_m_ exerting the strongest negative effect, whereas the effect of *l*_sc_ was relatively weak. In other words, compared with *l*_sc_, *l*_b_ (biochemical capacity) and *l*_m_ (*g*_m_) had relatively stronger influences on *A*_n_. Nevertheless, none of the coefficients reached the conventional significance threshold (*p* < 0.05), and the limited sample size (*n* = 3) meant that these results should be regarded as suggestive rather than conclusive. To further evaluate the combined effects of the three limitation components on *A*_n_, a multiple regression analysis was performed; its results were consistent with the simple regression and sensitivity analyses, again indicating that *l*_m_ had the strongest negative association with *A*_n_ (marginally significant), whereas *l*_b_ and *l*_sc_ had weaker positive effects, although none of the coefficients reached statistical significance at *p* < 0.05, probably owing to the limited sample size.

### 3.6. Effects of Soil N Application on Chlorophyll Content

N application significantly altered the leaf chlorophyll content, which followed a parabolic trend—initially increasing but then decreasing—with increasing N levels (Table 5). Compared with the CK treatment, all the N treatments significantly increased the chlorophyll a (Chl a), chlorophyll b (Chl b), and total chlorophyll (total Chl) contents (*p* < 0.05). Compared with the CK treatment, the MN treatment resulted in the highest chlorophyll contents, with the contents of Chl a, Chl b, and total chlorophyll increasing by 59.4%, 90.0%, and 66.7%, respectively. The chlorophyll levels in the LN group were intermediate, whereas those in the HN group slightly decreased, indicating that an excessive N supply does not further promote chlorophyll accumulation.

### 3.7. Effects of Soil N Application on Leaf N Content

The LNC was significantly affected by soil N application (Table 5). Compared with the control, the LNC increased by 36.3% in the low N application group (7.5 g urea m^−2^) and by 71.4% in the medium N application group (15.0 g urea m^−2^), reaching a maximum value of 44.9 ± 3.2 g kg^−1^ in the MN group. However, in the high N application group (22.5 g urea m^−2^), the LNC decreased to 37.1 ± 2.3 g kg^−1^, representing a 17.4% reduction relative to that in the MN group, but remained 41.6% higher than that in the CK group. Multiple comparisons revealed that the LNC in all N-treated groups was significantly greater than that in the CK group (*p* < 0.05). Notably, no significant difference was detected between the LN and HN groups (*p* > 0.05), whereas the LNC was greatest in the MN group, significantly differing from that in all the other treatment groups (*p* < 0.05).

### 3.8. Biochemical and Anatomical Traits Underlying Photosynthetic Limitations

To provide mechanistic insight into the shifts in photosynthetic limitation components, key biochemical and anatomical traits, including AQP activity; CA activity; Rubisco activity; and *T*_cw_, *S*_m_, *S*_c_, *g*_liq_, and *J*_f_, obtained from our companion study (Zuo et al., 2026) [18] were summarized in Table 6. Compared with the CK treatment, moderate N addition (≤15.0 g urea m^−2^) increased CA activity and Rubisco activity, with the highest values observed in the MN group (CA: 223.8 U L^−1^; Rubisco: 836.8 U L^−1^). However, in the HN group, Rubisco activity decreased to 807.4 U L^−1^, which was significantly lower than that in the MN group (p<0.05), whereas CA activity continued to increase to 238.3 U L^−1^. AQP activity differed among groups, being lowest in the LN group (604.0 U L^−1^) and highest in the HN group (678.3 U L^−1^). With respect to leaf anatomical traits, *T*_cw_ decreased from the CK group (0.36 μm) to the LN group (0.25 μm) but then increased in the HN group (0.35 μm), whereas *S*_m_ increased from the CK group (6.52 μm^2^) to the MN group (16.87 μm^2^) but decreased in the HN group (13.66 μm^2^). *S*_c_ did not significantly differ among the treatment groups. *g*_liq_ was highest in the MN group (4.27 × 10^−3^ mol CO_2_ m^−2^ s^−1^), which is consistent with the lowest *l*_m_ observed in the MN group.

These patterns were consistent with the changes in *l*_m_: the lowest *l*_m_ in the MN group coincided with the highest CA activity, Rubisco activity, *S*_m_, and *g*_liq_, whereas the sharp increase in *l*_m_ in the HN group coincided with decreased Rubisco activity and *S*_m_ and increased *T*_cw_. These results suggest that N-induced changes in biochemical capacity and leaf anatomy underlie the observed shifts in mesophyll limitation.

## 4. Discussion

Photosynthetic limitations are key determinants of plant photosynthetic efficiency, and their nature and magnitude are modulated by N supply levels [21,34]. Upon entering a plant, N is preferentially allocated to photosynthesis-related enzymes and electron transport components, thereby increasing the capacity for photosynthetic carbon assimilation [35,36]. The results of the present study revealed a nonlinear response of photosynthesis to N addition. Quantitative limitation analysis revealed that moderate N application (≤15.0 g urea m^−2^) progressively increased *l*_sc_ and *l*_b_ but significantly decreased *l*_m_ (see Figure 1). Consequently, *g*_sc_ and biochemical metabolic capacity (*V*_cmax_, which increased with increasing N supply, as shown in our previous study by Zuo et al. (2026) [18]), emerged as the dominant drivers of photosynthetic changes. These improvements were primarily attributed to increased activities of CA and Rubisco, as well as to favourable modifications in leaf anatomical traits—including reduced *T*_cw_, increased surface areas of mesophyll cells and chloroplasts exposed to intercellular airspaces per unit of leaf area (*S*_m_ and *S*_c_), and elevated liquid-phase diffusional conductance (*g*_liq_)—as detailed in our earlier study by Zuo et al. (2026) [18] (see Table 6). In contrast, a high N supply redirected leaf N partitioning from the photosynthetic apparatus towards storage and structural pools, thereby increasing *l*_m_ and suppressing photosynthesis [37]. Additionally, high N application reduced photosynthetic capacity by decreasing Rubisco activity and diverting leaf N into nonphotosynthetic storage pools [38]. Consistent with these reports, our study revealed that at 22.5 g urea m^−2^ (high N, HN), *l*_m_ increased rapidly and significantly surpassed both *l*_sc_ and *l*_b_ (see Figure 1), a pattern differing from those reported by some previous studies suggesting that excessive N primarily increases *l*_sc_ [15,39]. Instead, our results indicated that excessive N increased both *l*_m_ and *l*_b_. The concurrent decline in the LNC in the HN group (see Table 5) likely reduced substrate availability for CA and Rubisco synthesis, directly reducing *g*_m_ and carboxylation efficiency. Several factors likely contributed to this outcome: (i) nitrate accumulation in leaves damaged chloroplast structure, reducing *g*_m_; (ii) the synthesis and activity of photosynthesis-related enzymes (CA and Rubisco) were inhibited (see Table 6), lowering CO_2_ fixation efficiency; and (iii) an imbalanced leaf C/N ratio might have disrupted the transport and accumulation of photosynthetic products, further intensifying *l*_b_. In the HN group in the present study, *g*_m_ gradually surpassed *g*_sc_ and biochemical metabolic capacity as the dominant factors limiting photosynthesis (see Figure 2). This shift was attributed mainly to high-N-induced alterations in mesophyll cell structure and Rubisco activity. The increased *T*_cw_ and decreased *S*_m_ under high N application (see Table 6) not only lengthened the CO_2_ diffusion pathway [40] but also reduced the contact area between CO_2_ and chloroplastic carboxylation sites [23], directly decreasing the amount of CO_2_ available for carbon assimilation. Concurrently, the marked decline in Rubisco activity under high N application (see Table 6) substantially decreased carboxylation efficiency.

Appropriate N application (≤15.0 g urea m^−2^) increased the chlorophyll and leaf N contents (see Table 4 and Table 5) and subsequently increased Rubisco activity, decreased *l*_m_, and consequently improved photosynthetic efficiency. A clear parabolic response of the LNC to increasing N supply was observed; the maximum LNC was observed in the MN group (44.9 g kg^−1^), followed by a subsequent decline in the HN group (37.1 g kg^−1^) (see Table 5). This LNC trajectory closely mirrored the opposite trend in *l*_m_, which decreased progressively from the CK group to the MN group but then increased sharply in the HN group (see Figure 1), suggesting a mechanistic linkage between leaf N status and the efficiency of CO_2_ diffusion from the substomatal cavity to chloroplast carboxylation sites.

The LNC-dependent regulation of *l*_m_ can be attributed to at least three non-mutually exclusive mechanisms. First, increased LNC under moderate N supply promoted the synthesis of CA and AQPs (see Table 6), both of which are N-rich proteins that facilitate CO_2_ hydration and membrane transport, thereby increasing *g*_m_ (data available in our previous study by Zuo et al. (2026) [18]). Second, adequate leaf N availability supported the development of mesophyll anatomical traits—including by increasing *S*_m_ and reducing *T*_cw_—which collectively lowered the physical resistance to CO_2_ diffusion (see Table 6). Third, the decline in the LNC under excessive N application (HN) likely reflected a shift in N partitioning from the photosynthetic apparatus to nonphotosynthetic storage pools, leading to reduced *g*_m_ and consequently elevated *l*_m_ (see Figure 1). This interpretation is consistent with the strong negative correlation observed between *g*_m_ and *l*_m_ (r = −0.880, *p* < 0.001; Figure 3I) and the significant negative linear relationship between *A*_n_ and *l*_m_ (r = −0.776, *p* = 0.008; Figure 3II), reinforcing the view that *l*_m_ serves as the primary negative predictor of photosynthetic carbon assimilation under varying N supply. In contrast to its dominant effect on *l*_m_, the LNC showed a weaker direct relationship with *l*_sc_. Although moderate N addition increased both the LNC and *l*_sc_ (Figure 1), the contribution of *g*_sc_ to overall photosynthetic restriction remained relatively modest (*SC*_L_ ≤ 22%; Figure 2). These observations align with the finding that *l*_sc_ did not significantly linearly correlate with *A*_n_ (*p* > 0.05; Figure 3II), implying that *g*_sc_ was not a major limiting factor within the N gradient examined. Rather, the N-induced increase in *l*_sc_ might represent an incidental consequence of improved leaf structure and increased guard cell turgor [41,42] rather than a direct cause of photosynthetic enhancement. This interpretation is supported by the relatively low standardized coefficient of *l*_sc_ on *A*_n_ (beta = 0.261, *p* = 0.357; Table 4), indicating that stomatal factors played a subordinate role relative to mesophyll and biochemical constraints. Unlike *l*_m_ and *l*_sc_, *l*_b_ increased with N addition across the entire gradient (see Figure 1), although the magnitude of this increase was relatively small (*B*_L_ from 1% to 9%; Figure 2), and *l*_b_ exhibited a significant positive correlation with *A*_n_ (r = 0.686, *p* = 0.020; Figure 3II), suggesting that greater biochemical limitation was paradoxically associated with increased net photosynthesis—a counterintuitive finding that likely reflects a compensatory mechanism. The positive correlation between *A*_n_ and *l*_b_ should be interpreted with caution because *l*_b_ is a relative limitation index that is mathematically coupled with *l*_sc_ and *l*_m_. Under moderate N supply, the absolute biochemical capacity (Rubisco activity and electron transport rate) and *g*_m_ both increased, whereas the relative contribution of *l*_b_ to total limitation increased. This pattern reflects a coordinated adjustment among limitation components rather than evidence that *A*_n_ is unlimited by biochemical capacity. Under sufficient N conditions (≤15.0 g urea m^−2^), the increase in Rubisco activity and *J*_f_ (see Table 6) outweighs the concurrent increase in *l*_b_, resulting in a net increase in photosynthetic carbon assimilation [43,44]. This interpretation is consistent with the sensitivity analysis showing that *l*_b_ had the highest positive standardized coefficient for *A*_n_ (beta = 0.568, *p* = 0.063; Table 4), although this effect was only marginally significant. However, the marginal significance (*p* ≈ 0.06) suggested that the positive effect of *l*_b_ might be context dependent, varying with N availability and the prevailing physiological status of the leaf. Importantly, the decrease in the LNC in the HN group did not translate to a reduction in *l*_b_; instead, *l*_b_ continued to increase (see Figure 1). These results suggest that in the presence of excessive N, the biochemical machinery might be subjected to feedback inhibition or downregulation at the posttranslational level, even when the leaf N content remains relatively high (37.1 g kg^−1^). Such a decoupling between the LNC and *l*_b_ in the presence of high amounts of N highlights the limitation of using total LNC as a sole proxy for photosynthetic capacity and the importance of considering N allocation among photosynthetic components.

Specifically, correlation analyses between the limitation components and physiological traits (see Figure 3), together with sensitivity analysis of photosynthetic carbon assimilation (Table 1), revealed that *l*_sc_ and *l*_b_ exerted positive regulatory effects on net photosynthetic capacity, with *l*_b_ having the strongest positive effect and *l*_m_ having the most pronounced negative effect. Hence, the influence of photosynthetic limitation components on carbon assimilation capacity is environment dependent [45,46], with *l*_b_ (i.e., biochemical metabolic capacity) being the most sensitive variable, whose effect direction varies under different model conditions.

Nevertheless, despite the clear mechanistic insights obtained in this study, several caveats should also be noted. This study was conducted under greenhouse conditions with relatively stable temperature, light, and humidity, which may differ from field environments. In addition, we did not quantify nodule-related traits in the current experiment. Since mineral nitrogen fertilization may alter nodule formation and symbiotic nitrogen-fixation activity, future investigations should include quantitative measurements of nodule development to disentangle the interactive effects between exogenous mineral N supply and biological N-fixation in soybean. Moreover, the experiments were performed solely on soybean plants, and the regulatory effects of N application on photosynthetic limitations may vary across plant species. Third, the limitation analysis was inherently reference-dependent. The choice of CK as the reference state influenced the absolute values and potentially the ranking of limitation components. Although we conducted a reference-dependence sensitivity analysis (Section 3.4), the limited number of N levels (*n* = 4) and replicates (*n* = 3) constrained the statistical power of our conclusions. Future studies should include more N levels and larger sample sizes to validate the robustness of the inferred limitation rankings. Therefore, future studies should be carried out in the field using a broader range of species to validate the present findings and to provide a more comprehensive theoretical basis for optimizing soil N management strategies.

## 5. Conclusions

In this study, moderate soil N addition (≤15.0 g urea m^−2^) increased *l*_sc_ and *l*_b_ but markedly decreased *l*_m_, whereas excessive N addition (22.5 g urea m^−2^) led to a rapid increase in *l*_m_, which exceeded both *l*_sc_ and *l*_b_. The LNC showed a parabolic response to N supply, with a maximum at 15.0 g urea m^−2^ and a decline at 22.5 g urea m^−2^, and this pattern was associated with the observed shifts in the photosynthetic limitation components. The changes in net photosynthetic capacity were accompanied by altered activities of CA and Rubisco and by changes in leaf structure. These findings indicate that optimizing soil N addition could reduce *l*_m_ and that excessive N supply is associated with the greatest relative contribution of *l*_m_, but this conclusion requires validation by studies with larger sample sizes and alternative reference states. Further field studies involving multiple species are needed to validate these conclusions and to support rational N fertilization for sustainable agriculture.

## Figures and Tables

**Table 1:** Height (H) and base diameter (D) of the soybean seedlings.

Treatments	Height (H, cm)	Base Diameter (D, cm)
CK	35.3 ± 0.15 ^b^	1.0 ± 0.15 ^b^
LN	49.3 ± 0.12 ^a^	1.6 ± 0.12 ^a^
MN	48.1 ± 0.10 ^a^	1.6 ± 0.10 ^a^
HN	48.5 ± 0.15 ^a^	1.8 ± 0.15 ^a^

All the data are presented as the means ± SDs (*n* = 3). Different lowercase letters (a, b) in the same column indicate significant differences between N treatments at *p* < 0.05. CK, control; LN, low N addition; MN, medium N addition; HN, high N addition.

**Table 2:** Values of *αβ* for different N treatments.

Treatments	CK	LN	MN	HN
αβ	0.36 ± 0.02 ^b^	0.36 ± 0.01 ^ab^	0.38 ± 0.01 ^a^	0.37 ± 0.00 ^ab^

All the data are presented as the means ± SDs (*n* = 3). Different lowercase letters (a, b) in the same column indicate significant differences at *p* < 0.05. CK, control; LN, low N addition; MN, medium N addition; HN, high N addition.

**Table 3:** Relative contributions of photosynthetic limitation components to photosynthetic carbon assimilation calculated using the LN and MN treatments as alternative reference states.

Treatment	Relative Contribution (%) (LN as Alternative Reference)			Relative Contribution (%) (MN as Alternative Reference)		
	*SC*_L_	*MC*_L_	*B*_L_	*SC*_L_	*MC*_L_	*B*_L_
CK	5.92 ± 1.15 ^c^	12.01 ± 2.43 ^b^	4.61 ± 1.30 ^b^	3.19 ± 0.56 ^c^	19.29 ± 2.09 ^a^	6.29 ± 0.75 ^a^
LN	0 ^d^	0 ^c^	0 ^c^	8.81 ± 1.08 ^b^	9.01 ± 0.12 ^b^	5.40 ± 0.88 ^a^
MN	10.34 ± 1.28 ^b^	11.21 ± 1.69 ^b^	5.94 ± 1.46 ^b^	0 ^d^	0 ^c^	0 ^b^
HN	25.58 ± 2.90 ^a^	21.90 ± 2.95 ^a^	11.55 ± 1.47 ^a^	22.52 ± 2.27 ^a^	19.18 ± 2.13 ^a^	5.73 ± 0.87 ^a^

All the data are presented as the means ± SDs (*n* = 3). Different lowercase letters (a, b, c and d) in the same column indicate significant differences at *p* < 0.05. *SC*_L_, *MC*_L_ and *B*_L_ are the relative contributions of *g*_sc_ and *g*_m_ and biochemical capacity to d*A*_n_/*A*_n_, respectively.

**Table 4:** Linear regression analysis of the effects of photosynthetic limiting factors on photosynthetic carbon assimilation capacity.

Parameter	*A*_n_ (μmol CO_2_ m^−2^ s^−1^)					*V*_cmax_ (μmol CO_2_ m^−2^ s^−1^)				
	Beta	VIF	*p*	*F*-Value	df	Beta	VIF	*p*	*F*-Value	df
*l*_sc_ (%)	0.261	1.012	0.357	2.519	11	0.075	1.012	0.820	0.435	11
*l*_m_ (%)	−0.515	1.000	0.087	3.603	11	0.097	1.000	0.765	0.095	11
*l*_b_ (%)	0.568	1.012	0.063	2.519	11	−0.279	1.012	0.406	0.435	11

Beta is the standardized coefficient, VIF is the variance inflation factor, *p* is the significance coefficient, *p* < 0.05 indicates statistical significance, and df is the degree of freedom.

**Table 5:** Effects of different N treatments on the chlorophyll and leaf N contents of soybean plants.

Treatments	Chl a (mg g^−1^ FW)	Chl b (mg g^−1^ FW)	Total Chl (mg g^−1^ FW)	LNC (g·kg^−1^)
CK	3.2 ± 0.2 ^b^	1.0 ± 0.1 ^c^	4.2 ± 0.3 ^c^	26.2 ± 2.1 ^c^
LN	4.5 ± 0.2 ^a^	1.6 ± 0.2 ^b^	6.1 ± 0.4 ^b^	35.7 ± 1.8 ^b^
MN	5.1 ± 0.5 ^a^	1.9 ± 0.1 ^a^	7.0 ± 0.3 ^a^	44.9 ± 3.2 ^a^
HN	4.6 ± 0.4 ^a^	1.7 ± 0.1 ^ab^	6.3 ± 0.5 ^ab^	37.1 ± 2.3 ^b^

CK, control; LN, low N addition; MN, medium N addition; HN, high N addition; Chl a, chlorophyll a content; Chl b, chlorophyll b content; total Chl, total chlorophyll content; LNC, leaf N content. Different lowercase letters (a, b and c) in the same column indicate significant differences at *p* < 0.05.

**Table 6:** Values of key biochemical and anatomical traits in the different soil N addition treatment groups. All the data are presented as the mean ± SDs (*n* = 3). Different lowercase letters (a, b, c and d) indicate significant differences at *p <* 0.05. AQPs, aquaporins; CA, carbonic anhydrase; Rubisco, ribulose-1,5-bishosphate carboxylase; *T*_cw_, cell wall thickness; *S*_m_, surface area of mesophyll cells exposed to intercellular space per unit leaf area; *S*_c_, chloroplast surface area facing the intercellular space per unit leaf area; *J*_f_, photosynthetic electron transport rate; *g*_liq_, liquid-phase conductance to CO_2_. All the data presented in the table were obtained from our previous study (Zuo et al. (2026)) [18].

Treatments	AQPs Activity (U L^−1^)	CA Activity (U L^−1^)	Rubisco Activity (U L^−1^)	*T*_cw_ (μm)	*S*_m_ (μm^2^)	*S*_c_ (μm^2^)	*J*_f_ (μmol e^−^ m^−2^ s^−1^)	*g*_liq_ (10^−3^ mol CO_2_ m^−2^ s^−1^)
CK	643.7 ± 27.8 ^a^	198.8 ± 3.4 ^d^	690.5 ± 4.5 ^c^	0.36 ± 0.04 ^a^	6.52 ± 0.24 ^c^	3.69 ± 0.58 ^a^	131.5 ± 13.3 ^a^	3.10 ± 0.50 ^a^
LN	604.0 ± 13.0 ^b^	214.7 ± 3.3 ^c^	807.1 ± 3.4 ^b^	0.25 ± 0.03 ^b^	13.48 ± 2.47 ^b^	3.74 ± 0.91 ^a^	132.0 ± 15.9 ^a^	3.13 ± 0.80 ^a^
MN	644.7 ± 44.5 ^a^	223.8 ± 4.5 ^b^	836.8 ± 15.5 ^a^	0.28 ± 0.04 ^ab^	16.87 ± 0.08 ^a^	2.71 ± 0.50 ^a^	153.2 ± 12.2 ^a^	4.27 ± 0.84 ^a^
HN	678.3 ± 8.0 ^a^	238.3 ± 2.6 ^a^	807.4 ± 1.2 ^b^	0.35 ± 0.09 ^a^	13.66 ± 0.55 ^b^	3.28 ± 0.61 ^a^	157.0 ± 37.0 ^a^	3.54 ± 0.71 ^a^

## Data Availability

The datasets used and/or analyzed during the current study are available from the corresponding author upon reasonable request.
